# Activation *Versus* Inhibition of IGF1R: A Dual Role in Breast Tumorigenesis

**DOI:** 10.3389/fendo.2022.911079

**Published:** 2022-06-17

**Authors:** Joseph J. Bulatowicz, Teresa L. Wood

**Affiliations:** Department of Pharmacology, Physiology, & Neuroscience, Center for Cell Signaling and Cancer Institute of New Jersey, Rutgers Biomedical and Health Sciences, Newark, NJ, United States

**Keywords:** IGF1R, breast cancer, mammary gland, development, metastasis, differentiation

## Abstract

Historically, the body of literature surrounding the insulin-like growth factor type 1 receptor (IGF1R) has described a largely pro-tumorigenic role in breast cancer cells and in several transgenic or xenograft mouse models of breast cancer. Interestingly, however, more recent evidence has emerged that suggests an additional, previously undescribed, tumor and metastasis suppressive function for IGF1R in both human breast tumors and mammary oncogenesis in mice. These seemingly conflicting reports can be reconciled when considering what is currently known about IGF1R function in the context of tissue development and cancer as it relates to cellular growth, proliferation, and differentiation. In this mini review, we will summarize the currently existing data with a particular focus on mouse models that have been developed to study IGF1R function in mammary development, tumorigenesis, and metastasis *in vivo* and propose hypotheses for how both the tumor-promoting and tumor-suppressing schools of thought regarding IGF1R in these histological contexts are compatible.

## Introduction

The critical functions of the insulin-like growth factor (IGF)/IGF type 1 receptor (IGF1R) signaling axis in normal biological development (both systemic and tissue specific) have been extensively studied in several genetically altered mouse models. *In vivo* systemic deletion of IGF ligands or their receptors has resulted in related, but distinct, phenotypes exhibiting varying degrees of developmental impairment and/or lethality (1, 2). Perinatal lethality following systemic deletion of *Igf1r* necessitated the need for subsequent transplantation assays in order to define the role of the receptor in the mammary gland (3). Work performed with these models laid the foundation for the field’s current understanding of the importance of IGF1R function during embryogenesis and development of the mammary gland during puberty.

In addition to earlier studies focused on IGF1R developmental functions, more recent mouse models have been developed to investigate the receptor’s role in primary tumorigenesis. Consistent with the status of IGF1R as a receptor tyrosine kinase and its vigorously established function in promoting cell proliferation and survival, it was identified as a promising target for therapeutic intervention in human cancer patients. This led to the initiation of a number of clinical trials to disrupt IGF1R function in human tumors utilizing monoclonal antibody or small-molecule tyrosine kinase inhibitor-based therapies. Unfortunately, while early results were promising, the eventual conclusion from these trials was less than encouraging and, in some instances, led to worse outcomes denoted by systemic toxicity or worse patient prognosis [for reviews, see (4–7)].

In this mini review, we summarize the phenotypes of existing mouse models of modified IGF1R expression in mammary tissue (**Table 1**) and discuss observations made using human breast cancer data. We then attempt to reconcile these observations in order to shed light on the seemingly contradictory roles for IGF1R in breast cancer with a focus on mammary gland biology and tumorigenesis.

**Table 1:** Summary of the mouse models used to study the function of IGF1R in development and tumorigenesis.

Models of IGF1R Function in the Mammary Gland					
Genotype	Biological Context	IGF1R Status	Phenotype	Effect on Tumor Phenotype	Reference
*Igf1r^-/-^* systemic KO	Development	Deleted	Embryonic lethal, 45% normal birthweight, delayed bone/skin development	N/A	(1)
*Igf1r^+/+^*, *Igf1r^+/-^*, *Igf1r^-/-^* transplantation	Development	Deleted	Limited branch outgrowth and TEB formation during puberty	N/A	(3)
WAP-*dnIGF1R*	Pregnancy	Inhibited; mutated receptor	Decreased branching outgrowth, delayed alveolar density/differentiation during pregnancy	N/A	(8)
MMTV-*dnIGF1R*	Development	Inhibited; mutated receptor	Decreased post-pubertal branching, increased luminal progenitor and basal populations	N/A	(9)
MMTV-*CD8α*-*IGF1R*	Tumorigenesis	Constitutively activated	Induced tumorigenesis, increased luminal progenitor population	Promoting	(10)
MTB-*IGF1R*	Tumorigenesis	Overexpressed	Induced tumorigenesis	Promoting	(11)
Eef1a1-Kras^*^/WAP-*Cre*/*Igf1r^fl/fl^*	Tumorigenesis	Deleted	Increased tumor latency	Promoting	(12)
MMTV-*Wnt1*/*dnIGF1R*	Tumorigenesis	Inhibited; mutated receptor	Increased luminal progenitor and basal populations, decreased latency, increased metastasis	Suppressing	(9)
MMTV-*Wnt1*/K8-*CreER^T^*/*Igf1r^fl/fl^*	Tumorigenesis	Deleted	Increased luminal progenitor and basal populations, decreased latency, increased metastasis	Suppressing	(13)

N/A, Not Applicable.

## IGF1R in Mouse Mammary Gland Development

A number of techniques and mouse models (**Table 1**) have been developed to study the role of IGF1R in mammary gland development. Due to the immediate postnatal lethal phenotype exhibited by *Igf1r* ^-/-^ animals, alternative approaches were necessitated to study how loss of *Igf1r* influences mammary gland development (1). To bypass this technical limitation, pioneering experiments by Bonnette and Hadsell utilized tissue transplantation of mammary buds from *Igf1r^-/-^* embryos into host mice with mammary fat pads cleared of endogenous epithelium to examine epithelial growth during both puberty and pregnancy (3). Eight weeks post-transplantation, the *Igf1r* ^-/-^ transplanted animals had a significant decrease in the number of developed glands, as well as macroscopic abnormalities in ductal branching and terminal end bud (TEB) growth. Despite normal cellular organization of the ducts and TEBs in these animals, BrdU and TUNEL staining of 4-week post-transplantation mammary outgrowths revealed a significant decrease in proliferation and no evidence of cell death in TEB cells, specifically in the cap cell layer, which is responsible for most ductal outgrowth during puberty and harbors stem/progenitor populations necessary for formation of the ductal tree. This phenotype is strikingly similar to the developmental phenotype in the *Igf1* knockout mouse, where the number of TEBs and ductal expansion through the mammary fat pad was dramatically reduced, independently validating these observations (14). In contrast, mice with a heterozygous knockout of *Igf* had defects in alveogenesis during pregnancy, however, the lumens of preexisting alveoli were occluded with clusters of hyperproliferative epithelial cells (15). The phenotype observed in the *Igf1r^-/-^* transplantation model was partially rescued during pregnancy, where the pregnant *Igf1r* ^-/-^ transplanted animals exhibited a larger, hormone-induced fat pad outgrowth than wildtype transplanted mice relative to their virgin counterparts (3). This finding may be a result of a hypothetical decrease in dependence on IGF signaling and an increase in progesterone and prolactin signaling that takes place during the early stages of pregnancy and drives cellular proliferation and differentiation to fill the fat pad in preparation for lactogenesis (16, 17). Another potential explanation could be compensatory insulin receptor (INSR) signaling in the absence of *Igf1r* expression, supported by the observations that INSR substrates 1 and 2 undergo significant hormone-mediated changes during pregnancy (18).

To further define how IGF1R signaling influences mammary gland development during pregnancy, Sun *et al.* developed a model denoted as WAP-*dnIGF1R* (8). In this mouse model, the whey acid protein (WAP) promoter controls expression of a dominant-negative human IGF1R that is activated during mid-pregnancy at the onset of lactogenesis. These mice exhibited decreased alveolar outgrowth accompanied by a decrease in proliferation and no change in apoptosis (similar to the *Igf1r^-/-^* transplantation studies) suggesting the absence of required growth signals, i.e. IGF1 and IGF2 acting through the IGF1R. Additionally, these glands had alveolar differentiation as well as myoepithelial defects including a less elongated cellular morphology and a decrease in myoepithelial cell number as determined by reduced keratin (*Krt)14* expression (8). Consistent with these *in vivo* observations indicating that IGF1/IGF1R signaling functions in mammary epithelial cell differentiation, Merlo et al. showed that HC11 cells, an immortalized and undifferentiated mouse mammary epithelial cell line, can be induced to differentiate and activate milk protein gene casein (*Csn)2* expression *in vitro* using media containing prolactin, dexamethasone, and IGF1 (19, 20). These studies solidified the importance of IGF1R in normal mammary gland differentiation in addition to TEB cap cell proliferation during puberty.

Much of the subsequent studies and models developed to expand on the role of IGF1R in mammary gland biology were performed in the context of mammary carcinogenesis and, consequently, will be introduced and discussed partly in this section on IGF1R in mammary gland development and elaborated on in the next section. The first such model in order of publication was the MMTV-*CD8α-IGF1R* mouse (10). This line utilizes the mouse mammary tumor virus (MMTV) promoter that is highly active in mammary epithelial cells to express a constitutively active CD8α-IGF1R chimeric protein. The biochemical nature of the CD8α extracellular domain results in homodimerization after expression due to its affinity to form intramolecular disulfide linkages (10, 21). Homodimerization of the chimeric protein induces transphosphorylation and constitutive activation of the intracellular IGF1Rβ subunits. Whole mount staining of these glands during pubertal growth demonstrated an obvious phenotype of reduced TEB and fat pad outgrowth, defective ductal branching, and hyperproliferation of epithelial cells within the lumen of the ducts. As a result, the glands were morphologically dense and hyperplastic, resulting in tumorigenesis at about 8 weeks of age (10).

The MMTV-*CD8α-IGF1R* model addresses the role of constitutively active IGF1R in mammary gland development but may not recapitulate overexpression of IGF1R that would rely on endogenously expressed ligand activation. The MTB-*IGF1R* mouse is a mammary epithelium specific doxycycline-inducible IGF1R overexpression model that was created to investigate this gap in knowledge (11). Interestingly, this group found a similar developmental phenotype to the CD8α-IGF1R mice where ductal outgrowth was ablated and the tissue was densely clustered, hyperplastic, and hyperproliferative. This increase in proliferation was subsequently shown to be controlled by expression of cyclin D1 (22). As with the CD8α-IGF1R model, the hyperplasia eventually developed into palpable mammary tumors with an average latency of 71-78 days (11).

We also generated additional mouse lines to explore IGF1R function in both mammary gland development and tumorigenesis. The results of these studies yielded the MMTV-*dnIGF1R* line that expresses the same kinase-dead IGF1R mutant as the WAP animals referenced above (9). As with the gain of function models, this line with reduced IGF1R signaling makes use of the MMTV promoter that is activated in all mammary epithelial cells early in development. Post-pubertal glands expressing the *dnIGF1R* lacked extensive tertiary ductal alveolar budding at late pubertal stages after multiple estrous cycles, consistent with the *Igf1r* ^-/-^ transplantation studies showing reduced alveolar differentiation during pregnancy. Flow cytometry analyses of the MMTV-*dnIGF1R* post-pubertal glands revealed enriched luminal (Lin^-^CD24^+^CD29^low^) and luminal progenitor (Lin^-^CD24^+^CD29^low^CD61^+^) and decreased myoepithelial (Lin^-^CD24^+^CD29^high^) cell populations (9).

## IGF1R in Mouse Mammary Tumorigenesis

### Tumor Promoting Functions

A common conclusion of numerous published reports investigating IGF/IGF1R function in mammary tumorigenesis *in vivo* is that dysregulation of this pathway is sufficient to either induce tumorigenesis or to modulate the primary tumor phenotype (for summary, see **Table 1**). In the CD8α-IGF1R model, the authors described the primary tumors as highly proliferative and histologically homogeneous with areas of apparent necrosis (10). The high proliferation phenotype allowed the authors to culture primary cells and create xenograft models to determine the efficacy of IGF1R inhibitors on tumor cell growth. In this case, inhibition of IGF1R was sufficient to decrease proliferation, suggesting IGF1R as a potential target for chemotherapeutics (10). Farabaugh and colleagues continued to characterize this model and performed flow cytometry to investigate potential changes in epithelial lineages (23). Their findings revealed an increase in the basal population (Lin^-^CD24^+^CD29^high^CD61^+^) in the preneoplastic glands; however, this population was absent in CD8α-IGF1R tumors where, instead, the luminal progenitor population (Lin^-^CD24^+^CD29^low^CD61^+^) was increased (23). Furthermore, when these tumors were dissociated and subjected to *in vitro* differentiation assays, the resulting tumorspheres more closely resembled myoepithelial-like colonies, distinguished from their luminal counterparts by morphological analysis. This is consistent with the observations that luminal progenitors retain the capacity to differentiate into basal cells and further suggests an influence of IGF1R activation on mammary epithelial cell differentiation (24).

Perhaps not surprisingly, the MTB-*IGF1R* overexpression model showed a similar histological tumor phenotype to the CD8α-IGF1R model. Two tumor pathologies were described where smaller tumors histologically presented as solid sheets of cells with sparse extracellular space, similar to CD8α-IGF1R tumors, and larger tumors were more vacuous and likened to the phenotype of Wnt-driven mammary tumors (11). More recently, work in the MTB-*IGF1R* model revealed that expression of the microRNA cluster miR-200b/200a/429 suppresses tumor initiation driven by IGF1R overexpression although the intricacies of the mechanism remain unclear (25). Another, previously unmentioned, mouse mammary tumor model is the Eef1a1-Kras*/WAP-*Cre*/*Igf1r* ^fl/fl^ mouse line. This mouse line contains a mutated *Kras* gene including a premature stop codon, flanked by loxP sites for Cre recombinase recognition, under the control of a translational elongation factor promoter, Eef1a1, that is ubiquitously expressed in all cell types. Constitutive activation of Kras is controlled through tissue specific expression of Cre recombinase in order to remove the premature stop codon, resulting in constitutive Kras activation in a tissue of interest and subsequent tumor formation. Utilizing the WAP-*Cre* allowed the authors to study tumorigenesis specifically in pregnant mice. Tumors arising in these animals exhibit a basal-like gene expression signature in addition to upregulation of *Igf1r* expression, determined by microarray analysis, which led the authors to identify IGF1R as a viable therapeutic target. This model was developed as a proof-of-concept with the goal of inhibiting tumorigenesis in Eef1a1-Kras*/WAP-*Cre* animals. Conditional deletion of *Igf1r* significantly increased tumor latency in pregnant mice, reaffirming the status of IGF1R as an oncogene (12). These models provided strong *in vivo* evidence to support the conclusions that IGF1R is indeed pro-tumorigenic and has a particular role in promoting tumor cell proliferation.

### Tumor Suppressing Functions

In contrast to the above studies, other transgenic mouse lines exist that provide evidence supporting tumor and metastasis suppressive functions for the IGF1R (**Table 1**). We have generated novel transgenic mouse lines that alter either IGF1R function or expression in the context of Wnt1-driven tumorigenesis. Previously, we crossed the aforementioned MMTV-*dnIGF1R* line with the widely studied MMTV-*Wnt1* mammary tumor model, to generate a double transgenic animal, MMTV-*Wnt1*/*dnIGF1R*, to investigate the role of IGF1R signaling in a basal-like mouse model of breast carcinogenesis. Attenuating IGF1R signaling in this model resulted in a dramatic phenotype characterized by decreased tumor latency, increased tumor multiplicity, and a significant increase in lung metastasis in an otherwise low (<15%) metastatic tumor model, an observation that is unreported in the IGF1R overexpression models. These tumors have enhanced basal cell (Lin^-^CD24^+^CD29^high^) and luminal progenitor (Lin^-^CD24^+^CD29^low^CD61^+^) populations, suggesting inhibition of IGF1R interferes with differentiation or maintenance of a differentiated state (9). In addition to changes in cell population heterogeneity, these tumors have increased matrix metalloproteinase-secreting monocyte infiltration, collagen staining, as well as decreased epithelial adhesion originating from changes in cadherin expression (13, 26). Working with the MMTV-*Wnt1*/*dnIGF1R* animal model led to the question of cell lineage contribution to tumor initiation and metastasis. This resulted in the development of a novel system with a lineage-specific deletion of *Igf1r* in the context of Wnt-driven mammary gland tumorigenesis. The MMTV-*Wnt1*/K8-*CreER^T^*/*Igf1r^fl/fl^* line allows for investigation into the role of IGF1R specifically in the luminal lineage and to determine its effect on tumor phenotype. Similar to the dnIGF1R expressing Wnt tumors, luminal specific deletion of IGF1R resulted in lower tumor latency and increased metastasis compared to control animals (13). Work to fully characterize this model is still ongoing.

## IGF1R in Human Breast Cancer

Early studies investigating IGF1R expression in human breast cancer patients produced conflicting reports as to the prognostic value of IGF1R expression in patient samples (27–29). This was likely due to the varied methodologies employed in each study to determine expression, which was usually limited to immunohistochemical analysis of a small, finite number of relevant markers. In addition, the studies had relatively small sample sizes and lacked other relevant information, such as tumor molecular subtype. Microarray technology to evaluate gene expression in human breast cancer samples allowed researchers to identify gene expression profiles that characterized several genetically distinct tumor subtypes denoted as normal-like, luminal-A, luminal-B, HER2^+^, and triple-negative/basal-like (30, 31). In one of the earliest and largest (n = 2871) reports applying these criteria in conjunction with IGF1R expression data from human breast cancer patients, Yerushalmi *et al.* found a significant positive correlation between high IGF1R expression (IHC Allred score ≥ 7) and breast-cancer-specific survival (BCSS) in patients with luminal-B tumors. Conversely, high IGF1R expression was elsewhere associated with worse BCSS in patients with HER2/ERBB2-enriched tumors (32). Around the same time, other groups reported a strong positive correlation between IGF1R expression and patients harboring luminal type tumors and with BCSS (33–36). Additionally, a subsequent meta-analysis including data from these publications amongst others (10 studies, 5,406 patients) reiterated the findings that IGF1R expression levels positively correlated with overall survival and BCSS in hormone receptor positive tumors, but negatively correlated with survival in triple negative tumors (37). Taken together, these observations support the hypothesis that the role of IGF1R in the primary tumor phenotype is highly context dependent.

The relatively recent development of free, internet-based genomics tools to help facilitate advancement in cancer research has been become an invaluable resource. One such tool is the cBioPortal which functions to consolidate the ever-increasing number of publicly available human cancer datasets into one, easily searchable, user-friendly application (38, 39). To date, one such analysis through cBioPortal has been published employing The Cancer Genome Atlas (TCGA) RNA-sequencing breast cancer subset to further delineate correlations between IGF and insulin signaling molecule expression and PAM50 tumor molecular subtype (40–42). The major finding of this analysis, that included a number of molecules involved in these pathways was that IGF-related molecules are enriched on the transcriptional level in normal-like, luminal-A, and luminal-B tumors, and decreased in HER2^+^ and basal-like tumors, consistent with previous reports. Interestingly, INSR signaling has frequently been discussed as a possible compensatory mechanism for tumor cells when IGF1R is inhibited; however, these data suggest a positive correlation between IGF1R expression and INSR expression in human tumors. We performed a similar analysis for IGF1R expression using a different human breast cancer dataset, the Molecular Taxonomy of Breast Cancer International Consortium (METABRIC) database, with a focus on correlations between IGF1R expression and PAM50 subtype, as well as probability of survival (43, 44). This analysis further confirmed a positive association with IGF1R expression and hormone receptor positive tumors and a negative correlation with triple-negative tumors. Importantly, high IGF1R expression was associated with a better probability of survival, regardless of hormone receptor status (26).

**Figure 1:**
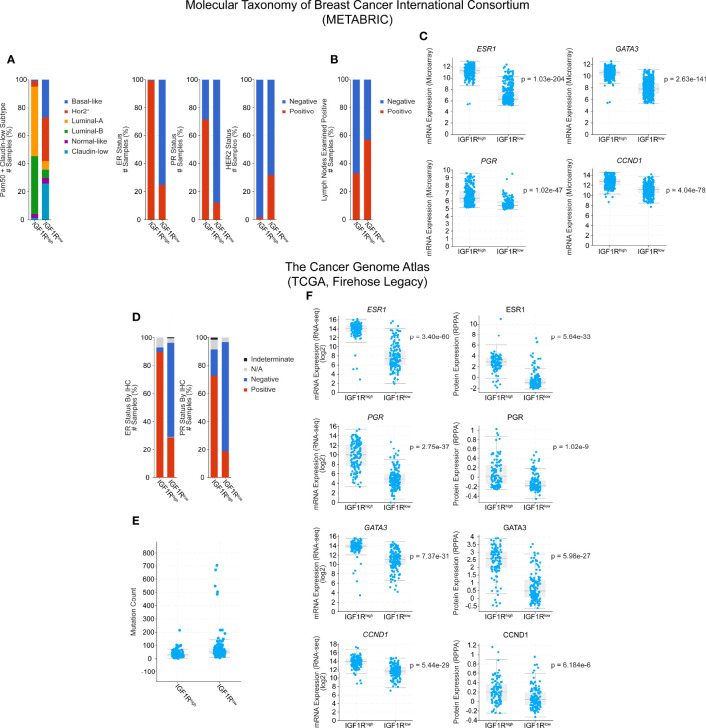
Clinical characteristic and gene expression analysis of IGF1R^high^ and IGF1R^low^ human breast tumors from the Molecular Taxonomy of Breast Cancer International Consortium (METABRIC) and The Cancer Genome Atlas (TCGA) databases. Cohorts were generated by classifying patients by either high (z-score > 1, METABRIC n = 300, TCGA n = 128) or low (z-score < -1, METABRIC n = 415, TCGA n = 153) expression of IGF1R, while average patients (-1 < z-score < 1) were excluded. **(A)** PAM50 classification of patient tumors and hormone receptor expression status. **(B)** Lymph node positivity. **(C)** Gene expression microarray data for *ESR1*, *PGR*, *GATA3*, and *CCND1*. **(D)** Hormone receptor status in the TCGA cohorts. **(E)** Mutation count. **(F)** Gene (left) and protein (right) expression data for *ESR1*, *PGR*, *GATA3*, and *CCND1*. Statistical analyses were generated by cBioPortal and are student t-tests, where significance was defined as p < 0.05. N/A, Not Available.

Here, we extend these analyses by using cBioPortal to further explore the human data in both TCGA (TCGA Firehose Legacy) and METABRIC databases. Narrowing the available patient set to only include tumors which express higher than average IGF1R (z-score > 1) and lower than average IGF1R (z-score < -1), we see a shift in the PAM50 subtypes where IGF1R^high^ tumors are more commonly luminal-A and luminal-B and IGF1R^low^ tumors are classified more commonly as basal, ERBB2^+^/HER2^+^, or claudin-low (**Figure 1A**). Additionally, in both the METABRIC and TCGA subsets, there are lower levels of hormone receptor expression in the IGF1R^low^ cohort compared to IGF1R^high^ as well as a correlation with HER2^+^ tumor classification (**Figures 1A, C, D, F**), similar to the findings of Farabaugh et al. (41). Interestingly, lymph node positivity, a readout of early-stage metastasis, is ~20% higher in the METABRIC IGF1R^low^ group, providing a human correlation between low IGF1R levels and metastasis, consistent with our previous observations in the MMTV-*Wnt1/dnIGF1R* model [**Figure 1B** (9)]. In the TCGA dataset, these observations are consistent and extend beyond the level of transcription with the protein expression data that is also available for each tumor sample (**Figure 1F**, right). Mutational load was another clinical characteristic that was altered between the groups with IGF1R^low^ patients having a higher mutational burden (**Figure 1E**). Expression of CCND1 in both datasets is increased in the IGF1R^high^ groups and recapitulates the findings from the MTB-*IGF1R* model that tumorigenesis resulting from IGF1R overexpression is cyclin D1-driven [**Figures 1C, F** (22)]. Furthermore, expression of IGF1R is also positively correlated with GATA3, a well characterized promoter of luminal lineage differentiation and whose loss of expression is associated with enrichment of the luminal progenitor population [**Figures 1C, F** (45, 46)].

## Discussion

The field’s understanding of the role of IGF1R in breast cancer has continued to evolve over decades of study. Early work summarized above convincingly justified the classification of IGF1R as an oncogene with potent value as a therapeutic target in human patients. More recently, though, following the failure of the many clinical trials initiated with the goal of inhibiting IGF1R in human patients, and recent data illustrating the effect of inhibiting IGF1R on enhancement of metastasis, it has become clear that the receptor’s role in tumorigenesis is more nuanced and complicated than previously thought. This is also reflected in the literature by a number of studies that attempt to identify different mechanisms of compensation induced by IGF1R inhibition and the efficacy of a dual inhibitory approach with drugs such as cisplatin, trastuzumab, and others (47–51). Conversely, in some contexts, it may be beneficial to subsequently target the IGF pathway in situations where upregulation or activation is observed secondary to administration of therapy (52). Importantly, however, the mechanistic questions still remain as to how both driving and blocking IGF signaling *via* IGF1R result in a tumor promoting phenotype.

Constitutive activation or overexpression of IGF1R is sufficient to induce tumorigenesis characterized by tumors with an increased luminal progenitor population (11, 23). Previous work from our lab utilizing the MMTV-*Wnt1/dnIGF1R* mouse tumor model also observed a similar increase in the tumor luminal progenitor population (9). This seemingly contradictory observation could potentially be explained when considering the different biological processes in which IGF1R plays a role, context (or cell type/stage) specificity, and the unlikely compatibility of data from many of the models outlined above. In the case of the MMTV-*CD8α*-*IGF1R* model, it is feasible to hypothesize that constitutive activation of IGF1R is driving proliferation of the luminal progenitors at the adolescent stage prior to the onset of puberty, since this is the developmental stage during which the MMTV-LTR activates (53). This is further supported by the data demonstrating that these mice have stunted ductal outgrowth accompanied by hyperproliferation of epithelial cells within the lumens of the rudimentary ductal tree, as well as similar developmental defects also observed in the MTB-*IGF1R* overexpression model. This suggests that the cell-of-origin for the CD8-IGF1R tumors is possibly a luminal progenitor cell whose proliferation may be driven early in development through IGF1R signaling.

On the other hand, the shift seen in the luminal progenitor population of the MMTV-*Wnt1/dnIGF1R* model could be attributed to both the fact that the tumors are formed as a result of *Wnt1* overexpression [which could potentially drive progenitor cell expansion (54)] and the strongly supported role of IGF1R in luminal lineage differentiation. Early work in the MMTV-*Wnt1* model has shown that these tumors express both *Krt6* and *Sca1*, markers for mammary progenitor cells that are not expressed in tumors arising from MMTV-*Neu* or MMTV-*PyMT* animal, suggesting a role of progenitors in initiation of Wnt-driven tumors (54). Tumors resulting from the MMTV-*Wnt1* mouse tumor model are phenotypically basal-like, and historically, basal-like tumors were hypothesized to originate from a transformed myoepithelial progenitor cell (55). However, Molyneux *et al.* demonstrated that basal-like tumors resulting from BRCA1 mutations are derived from luminal progenitors, and not myoepithelial cells (24, 56). Similar to the MMTV-*Wnt1* mouse, tumors containing BRCA1 mutations have a significant population of luminal progenitors. Additionally, BRCA1 plays a role in the DNA damage response, a process that IGF1R has also been shown to positively regulate, suggesting inhibition of BRCA1 could potentially result in a similar phenotype as inhibition of IGF1R [**Figure 1E** (47, 57, 58)]. This mechanism could hypothetically be influenced by a decrease in IGF1R signaling resulting in a block of luminal lineage differentiation while concomitantly hampering the DNA damage response, driving accumulation of luminal progenitors, and increasing the statistical odds of tumor initiation in this population as a result of an increase in mutational burden, especially in the context of Wnt1-driven proliferation.

Another important piece of data unique to the MMTV-*Wnt1/dnIGF1R* model was an observed shift in insulin receptor isoform expression. The gene expression ratio of INSR-A to INSR-B is significantly higher in these tumors and is of importance due to the high affinity of IGF2 for INSR-A, identifying one potential mechanism of resistance to IGF1R inhibition (9). Critically, a similar correlation was seen in human participants of at least one unsuccessful IGF1R-targeting clinical trial where patients, regardless of treatment group, with higher expression levels of INSR-A or INSR-B had significantly shorter progression-free survival (59). These observations serve to further support the translational relevance of the MMTV-*Wnt1/dnIGF1R* model to human disease.

An important distinction between the overexpression/constitutive activation and inhibition models is the fact that tumors arising from inhibition of IGF1R are metastatic, while the existence of metastases in the former has not been reported (9). This is particularly of interest considering metastasis is the overwhelming cause of death in cancer patients (60). A recent study of breast cancer patients published in 2017 revealed a correlation between metastasis and low levels of IGF1R in isolated circulating tumor cells, further supporting the metastatic phenotype seen in our model and the human METABRIC data [**Figure 1B** (61)]. In conclusion, the studies summarized in this mini review highlight the clinical relevance of contextual IGF1R expression during breast cancer tumorigenesis and emphasize the need for further research in order to more thoroughly define the mechanisms distinguishing IGF1R^high^ and IGF1R^low^ tumors with the ultimate goal being more targeted and effective therapeutic strategies for patients.

## Author Contributions

JB wrote the manuscript, performed the database analyses, and generated the figure and table. JB and TW contributed to the manuscript revision, read, and approved the submitted version.

## Funding

This work was supported by Public Health Service National Institutes of Health grants NCI R01CA204312 (TW).

## Conflict of Interest

The authors declare that the research was conducted in the absence of any commercial or financial relationships that could be construed as a potential conflict of interest.

## Publisher’s Note

All claims expressed in this article are solely those of the authors and do not necessarily represent those of their affiliated organizations, or those of the publisher, the editors and the reviewers. Any product that may be evaluated in this article, or claim that may be made by its manufacturer, is not guaranteed or endorsed by the publisher.
